# Optimization of Cadmium Adsorption by Magnetic Graphene Oxide Using a Fractional Factorial Design

**DOI:** 10.3390/ijerph17186648

**Published:** 2020-09-11

**Authors:** Hui Wang, Yiming Zhou, Xinjiang Hu, Yuan Guo, Xiaoxi Cai, Chunjie Liu, Ping Wang, Yunguo Liu

**Affiliations:** 1 College of Environmental Science and Engineering, Central South University of Forestry and Technology, Changsha 410004, China; wanghui@csuft.edu.cn (H.W.); 20181200405@csuft.edu.cn (Y.Z.); xjhu@csuft.edu.cn (X.H.); 2 Faculty of Life Science and Technology, Central South University of Forestry and Technology, Changsha 410004, China; 3 Institute of Bast Fiber Crops, Chinese Academy of Agricultural Sciences, Changsha 410205, China; guoyuan@caas.cn (Y.G.); liuchunjie@caas.cn (C.L.); 4 College of Art and Design, Hunan First Normal University, Changsha 410205, China; xiaoxi@hnu.edu.cn; 5 College of Environmental Science and Engineering, Hunan University, Changsha 410082, China

**Keywords:** magnetic graphene oxide, removal, cadmium, fractional factorial design, optimization

## Abstract

Graphene materials have attracted increasing interest in water remediation. In this study, magnetic graphene oxide (MGO) was prepared through the modified Hummers method and the adsorption behaviors of cadmium were investigated. Firstly, the sorption kinetics, isotherms, as well as the effects of pH were investigated. Then, fractional factorial design (FFD) was used to optimize the effects of pH, temperature, time, initial concentration of cadmium ion and NaCl on cadmium adsorption. The results indicate that MGO could effectively remove cadmium ions from an aqueous solution and the sorption data could be described well by pseudo-second-order and Freundlich models, showing that the adsorption rate of cadmium ions on MGO is multilayer adsorption and dominated by the chemical adsorption. According to the FFD results, the maximum adsorption capacity of cadmium ions was 13.169 mg/g under the optimum condition of pH value 8, 45 °C, contact time 60 min, initial cadmium concentration of 70 mg/L and NaCl concentration of 100 mg/L. Higher levels of the pH value, temperature and initial cadmium concentration are beneficial to the adsorption process. These results are important for estimating and optimizing the removal of metal ions by MGO composite.

## 1. Introduction

In recent years, with the rapid development of industry, arbitrary discharge of waste water has caused serious heavy metal pollution. It also poses a serious threat to the health of humans and other creatures [1]. Cadmium is a very toxic heavy metal and is widely used in electroplating, coating and metallurgy industries [2]. Cadmium can enter the body through respiratory and digestive systems, endangering human health [3]. Various diseases such as emphysema, kidney damage, diabetes, cardiovascular disorders and bone malformations are associated with cadmium ion hazards [4,5,6]. In addition, cadmium ions also have toxic effects on plants [7]. Cadmium that is absorbed by plants can destroy various dehydrogenases of plants, thus inhibiting plant growth and even causing plant death. The use of water contaminated with cadmium to irrigate crops will cause cadmium to accumulate in agricultural crops, causing the cadmium enriched in crops such as wheat, soybeans and rice to exceed standards, and then threatening human health through the food chain [8].

Since cadmium ions have many harmful effects on the human body, animals and plants, and cannot be easily degraded in nature, we need to find a suitable treatment. In general, there are many methods for removing heavy metal ions in aqueous solutions, such as coprecipitation, biosorption, ion exchange, membrane separation, and adsorption [9,10]. Among them, the adsorption method has the advantages of simple operation, good treatment effect and low cost, is one of the most important water treatment methods and is widely used in the field of wastewater treatment [11,12]. Many materials such as silica, titanium dioxide (TiO_2_), montmorillonite, chitosan, kaolinite and magnetic particles have been used as adsorbents [13,14,15]. However, the traditional adsorbent has low adsorption capacity, and it is difficult to realize solid and liquid separation after adsorption. Therefore, it is urgent to develop new and efficient adsorbents to overcome the inherent defects of conventional adsorbents.

Graphene is a single-layer honeycomb lattice structure formed by the hybridization of carbon atoms via sp^2^ electron orbit [16,17,18,19]. The large specific surface area makes graphene a good adsorption material [20]. However, there is a strong interaction between graphene sheets, which results in inactive surface chemistry and makes the dispersion of graphene in aqueous solution poor and easy to agglomerate, affecting its adsorption performance, and eventually limiting the application of graphene in wastewater treatment [21]. Graphene oxide (GO) is an oxide of graphene. It is produced by oxidizing exfoliated graphite by a strong oxidant [22]. Its surface is rich in oxygen-containing groups, including hydroxyl groups, epoxy groups, and carboxyl groups [23]. These functional groups provide the basis for a high-performance adsorption material for GO. In the previous study, we found that the oxygen-containing groups on the surface of the GO have hydrophilic properties and are not easily separated from the solution after adsorption [11]. Hence, a magnetic graphene oxide (MGO) may be prepared by coupling magnetic nanoparticles with GO, which may effectively remove pollutants from water and achieve rapid and efficient separation under the action of an external magnetic field.

Usually, the effects of different factors on the adsorption process are different [24,25,26]. Conventional studies on optimizing adsorption conditions alone separately would take a long time. In this study, the fractional factorial design (FFD) was used to optimize the effects of the interaction on cadmium adsorption. The application of this method will be more effective and comprehensive.

The present work is focused on: (1) synthesizing an easily separated MGO composite; (2) examining the sorption capacity of MGO to cadmium ions in aqueous solution; (3) investigating the effects of pH, time, initial concentration, temperature and other conditions on the cadmium ions adsorption of MGO; (4) exploring the adsorption mechanism of MGO on cadmium ions by kinetic and isotherm models; (5) studying the main effect and interaction effect of various factors on the cadmium ion adsorption process using FFD.

## 2. Materials and Methods

### 2.1. Material

Graphite powder was purchased from Tianjin Hengxing Chemical Preparation Co., Ltd. (Tianjin, China) Sodium formate, sodium benzoate, and sodium chloride were provided by Sinopharm Chemical Reagent Co., Ltd. (Shanghai, China). All chemicals used in this research were of analytical grade and underwent no further purification during the entire process. Deionized water (DW) was used to prepare the desired solutions in all the experiments. MGO was synthesized from natural graphite powder following a modified Hummers method according to previous studies [2,17].

### 2.2. Characterization of MGO

The surface morphology and elemental composition of the MGO was investigated by scanning electron microscopy (SEM, Quanta−200, The Netherlands). Energy dispersive spectroscopy (EDS) spectra were measured by an energy-dispersive X-ray spectrometer (EDAX Genesis 2000, USA). X-ray diffraction (XRD) patterns of MGO were obtained by using a powder diffractometer (Rigaku D/max−2500) with a Cu Kα source. A Fourier transform infrared (FT-IR) spectroscopy (PerkinElmer S pectrum One, Waltham, MA, USA) was applied to characterize the samples by using the KBr pellet technique. The X-ray photoelectron spectroscopy (XPS) was performed on an ESCALAB 250Xi X-ray photoelectron spectrometer (Thermo Fisher, Waltham, MA, USA).

### 2.3. Adsorption Studies

The Cd(II) stock solution (1000 mg/L) was prepared by dissolving 1.000 g pure cadmium powder in 20 mL HNO_3_ solution (20%) in a 1000 mL flask, with Milli-Q water added up to the mark. Working solutions were prepared by appropriate dilution of stock solution using Milli-Q water. All adsorption experiments were performed by adding MGO suspensions to achieve the required adsorbent concentration, and performed in a constant temperature shaker with a rotating speed of 180 rpm. The solution pH was adjusted using 0.01 mol/L NaOH or 0.01 mol/L HCl [27]. After 24 h of adsorption, MGO was separated from the solution with magnets. The Cd(II) concentration in the supernatant was measured using flame atomic adsorption spectrometry (PerkinElmer AA700, USA) [28]. All trials were in triplicate. The adsorption capacity (*q*_e_) of MGO was calculated by the following formula:

*q*_e_ = (*C*_0_ − *C*_e_) × *V*/*m* (1)

where *C*_0_ and *C*_e_ are the initial concentrations and equilibrium concentration of cadmium (mg/L), respectively. *V* is the volume of the suspension and *m* is the mass of MGO.

### 2.4. Optimization of the Adsorption Conditions

The adsorption experiments were conducted based on a fractional factorial design (FFD) to evaluate the effects of process variables including pH value, temperature, time, initial concentration of cadmium ions and NaCl concentration. A 2^5−1^ fractional factorial design (FFD) with a resolution of *V* was developed. Table 1 shows the level of each factor above, and Table 2 describes the 2^5−1^ FFD. Here, FFD was established by Minitab software (Minitab Inc, version 16) and the experimental data were analyzed.

## 3. Results and Discussions

### 3.1. Characterization

**Figure 1:**
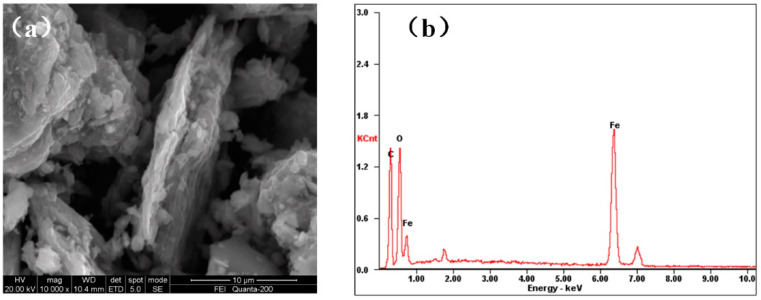
Characterization of magnetic graphene oxide (MGO): (**a**) SEM image; (**b**) EDS analysis.

Figure 1a shows the SEM figure of MGO. According to Figure 1a, after ferro ferric oxide is loaded onto the surface of GO, some graphene oxide sheets are aggregated. This causes a loss to some adsorption sites, thus affecting the adsorption effect against heavy metal ions. Figure 1b shows the energy spectrum diagram about MGO. Compared with the GO specified in the previous chapter, the GO here contains a large quantity of Fe element, which is mainly available from the Fe_3_O_4_ magnetic nanoparticles deposited on the surface of GO.

**Figure 2:**
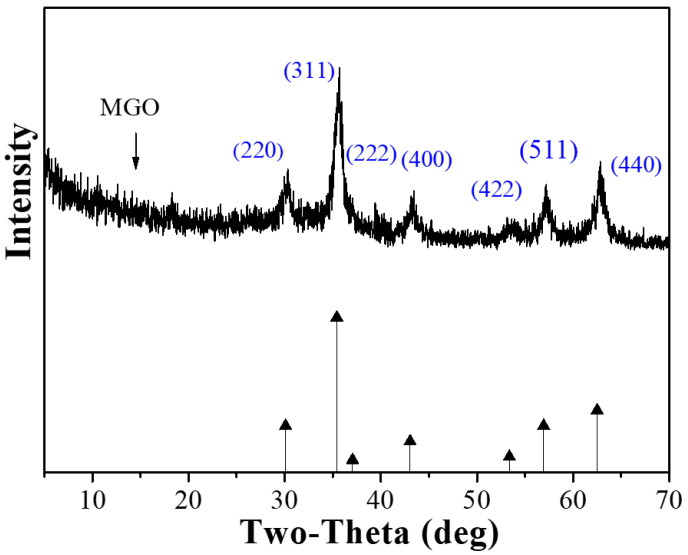
XRD patterns of MGO.

Figure 2 shows the results of XRD analysis when the 2*θ* of MGO ranges from 20° to 70°. The characteristic peaks available at the 2*θ* of 30.1°, 35.2°, 37.1°, 43.1°, 53.4°, 56.9° and 62.5°, respectively, correspond to the position index of (220), (311), (222), (400), (422), (511) and (440) [29], thus revealing obvious characteristics of iron oxides (JCPDS Card No. 19−0629). This shows that the Fe_3_O_4_ magnetic nanoparticles are successfully loaded onto the surface of graphene oxide. Compared with the XRD diagram on GO specified in the previous research [9], no characteristic peak of GO at the 2*θ* of 11.5° is detected in the XRD diagram on MGO. This is mainly due to the fact that Fe_3_O_4_ nanoparticles mainly exist between graphene oxide sheets, thus destroying the stacking structure of graphene oxide sheets [30,31].

**Figure 3:**
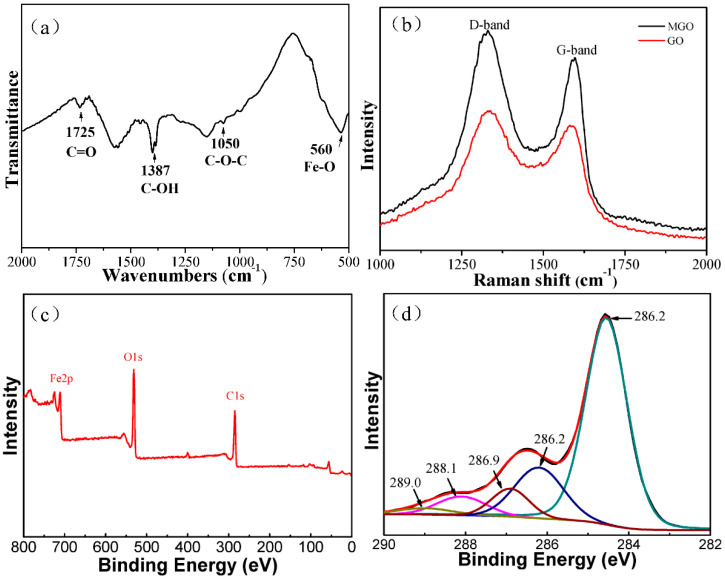
Characterization of MGO: (**a**) FT-IR spectrum; (**b**) Raman spectra; (**c**) XPS survey scan spectrum; (**d**) C1s XPS spectrum.

Figure 3a shows the infrared spectrogram (FT-IR) of magnetic graphene oxide. In this figure, the characteristic peak at 1725 cm^−1^ is the characteristic peak of C = O in carboxyl and carbonyl groups, the characteristic peak at 1387 cm^−1^ is the stretching vibration peak of C-OH in carbonyl groups, the characteristic peak at 1050 cm^−1^ is the stretching vibration peak of C-O-C in epoxy groups, and the characteristic peak at 560 cm^−1^ is the characteristic peak of Fe-O in Fe_3_O_4_. The above analysis results show that there are still a variety of active groups on the surface of MGO, and Fe_3_O_4_ nanoparticles are also loaded successfully onto the surface of GO [24].

Figure 3b shows the Raman spectrum diagram about MGO. For magnetic graphene oxide and graphene oxide, there are obvious characteristic peaks at the positions of 1332 cm^−1^ and 1581 cm^−1^, indicating a good bonding of Fe_3_O_4_ nanoparticles and graphene oxide. The two characteristic peaks represent sections D and G, respectively. Peak G near to the position of 1581 cm^−1^ is the characteristic peak of sp^2^ of carbon structure, reflecting its symmetry and degree of crystallization; Peak D near to the position of 1332 cm^−1^ is a defect peak, reflecting the disorderliness and defect level of graphene sheets. Usually, the intensity ratio of Peak D to Peak G (*I*_D_/*I*_G_) can be used to measure the size and defect density of the graphene conjugate plane. The *I*_D_/*I*_G_ value of magnetic graphene oxide is higher than that of graphene oxide; this is possibly due to the fact that Fe_3_O_4_ nanoparticles are introduced to the carbon sp_2_ orbit of graphene oxide [32].

As shown in Figure 3c, MGO has three obvious peaks, which are associated with C1s, O1s and Fe2p respectively. Figure 3d shows the XPS diagram of C1s. In the magnetic graphene oxide, carbon atoms are bonded with other elements in five forms: C-C (284.6 eV), C-O (286.2 eV), C-O-C (epoxy group, 286.9 eV), C = O (carbonyl, 288.1 eV) and O-C = O (289.0 eV) [25,33,34]. Therefore, certain active groups are nevertheless retained on the surface of GO, although Fe_3_O_4_ magnetic nanoparticles are loaded on the surface of GO.

### 3.2. Effect of pH

**Figure 4:**
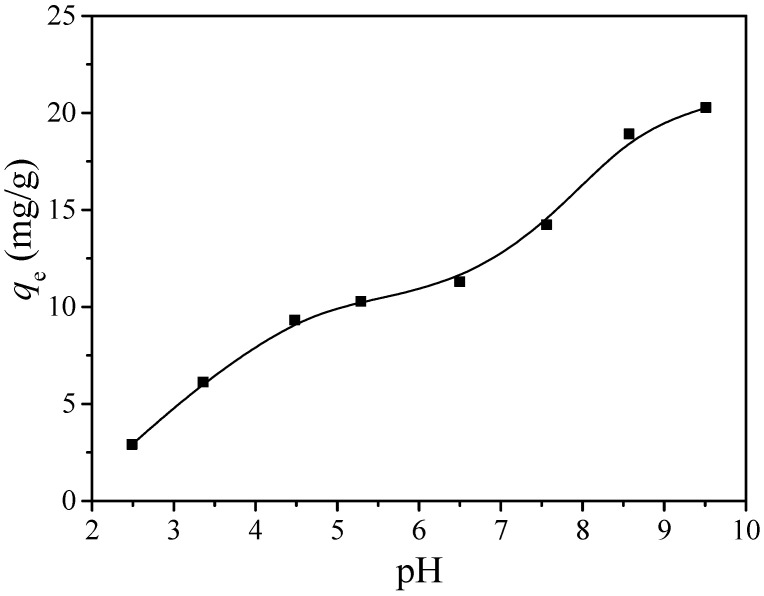
Effect of the solution pH on Cd(II): *m*/*V* = 84 mg/L, *t* = 24 h, *C*_0(Cd)_ = 10 mg/L, *T* = 30 °C.

In general, the pH range of wastewater is wide. In the process of the adsorption reaction, the solution pH is one of the most important influence factors, and it will affect the combination of heavy metal ions and the surface of the adsorbent [35], because the solution pH can affect the adsorption properties of chemical properties and the binding sites on the surface of the adsorbent. Therefore, in the process of wastewater treatment, the influence of the pH value on the adsorption process needs to be considered. Figure 4 shows the change in the adsorption capacity of MGO on cadmium ions when the pH is from 2.5 to 9.5. The results indicate that with the increase in solution pH, the cadmium ions’ adsorption capacity also gradually increases; this suggests that the pH has a great influence on the adsorption process. According to our previous work [2], the main existing forms of cadmium ions in different pH values are Cd^2+^, Cd(OH) ^+^, Cd(OH)_2_, Cd_2_(OH)^3+^, Cd(OH)_3_^−^ and Cd(OH)_4_^2−^. While pH < 8, the main existing form of cadmium ion is Cd^2+^. The zeta potential of the MGO was measured to be 4.2. This means that the MGO surface is positively charged at solution pH < 4.2, and negatively charged at pH > 4.2. Hence, adsorption when pH > 4.2 is more likely to occur between the positively charged cadmium (Cd^2+^) ions and negatively charged surface. So, as pH increases, the amount of cadmium adsorption will continue to increase. However, when the solution pH > 8, the cadmium ions in the solution begin to form precipitation, which may affect the calculation results of the adsorption process.

### 3.3. Adsorption Kinetics

**Figure 5:**
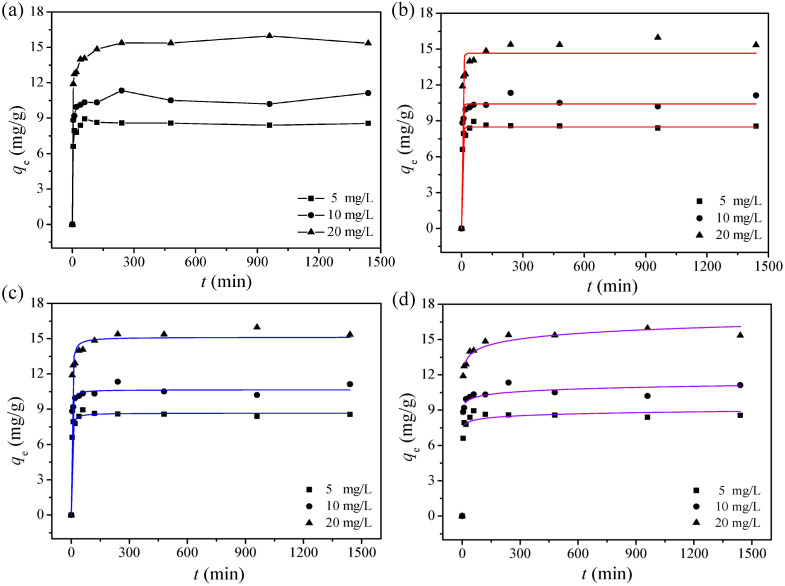
(**a**) Time profiles of Cd(II) adsorption with MGO; (**b**) pseudo-first-order; (**c**) pseudo-second-order; (**d**) Elovich model: *m*/*V* = 84 mg/L, pH = 6.00 ± 0.01, *C*_0(Cd)_ = 10 mg/L, *T* = 30 °C.

Adsorption rate is one of the most important parameters for evaluating the efficiency of an adsorption process [36]. In order to understand the adsorption mechanism of cadmium ions on MGO, we analyzed the adsorption kinetics of MGO for three different cadmium ion concentrations. The results are shown in Figure 5a. It was found that MGO can adsorb cadmium ions at a very high rate, where the adsorption equilibrium was reached within 1 h. The experimental data were further fitted using the pseudo-first-order, pseudo-second-order, and Elovich kinetic models, as shown in Figure 5b–d, respectively. The corresponding parameters used in these fittings are summarized in Table 3. Combining these fitting results with the calculation results, it can be seen that the pseudo-second-order yields the highest *R*^2^ value. In addition, the maximum adsorption capacity predicted by the pseudo-second-order kinetic model for cadmium ion concentrations of 5, 10, and 20 mg/L are 8.667, 10.65, and 15.13 mg/g, respectively. These values are closest to the experimental data among those predicted by the different models. This finding suggests that the pseudo-second-order model is the most appropriate for describing cadmium ion adsorption on MGO. Furthermore, the experimental results confirm the assumption made in the pseudo-second-order model. It can be stated that the most probable mechanism is chemical sorption involving adsorbent–adsorbate electron exchange [37]. The initial adsorption rate *h* can be calculated according to the constant *k*_2_ used in the pseudo-second-order model. Based on the results shown in Table 3, the value of *h* for the three different cadmium concentrations (5, 10, and 20 mg/L) was 6.235, 8.734, and 8.699 mg/g min, respectively. This shows that a higher initial cadmium ion concentration can yield a higher initial adsorption rate. However, when the initial concentration reached 20 mg/L, the reaction rate decreased slightly. This phenomenon can be explained by the following effects. When the cadmium ion concentration is very small in the solution, the collision frequency between the cadmium ions and the MGO is quite low at the adsorption sites. With increasing cadmium ion concentration, there is a higher possibility for the ions to collide with the MGO at the adsorption sites, and the reaction rate increases. With a further increase in cadmium ion concentration, the high-energy adsorption sites on the MGO surface become saturated. Therefore, the cadmium ions can now only adsorb on the low-energy sites, and this results in a lower reaction rate [38,39].

### 3.4. Adsorption Isotherms

**Figure 6:**
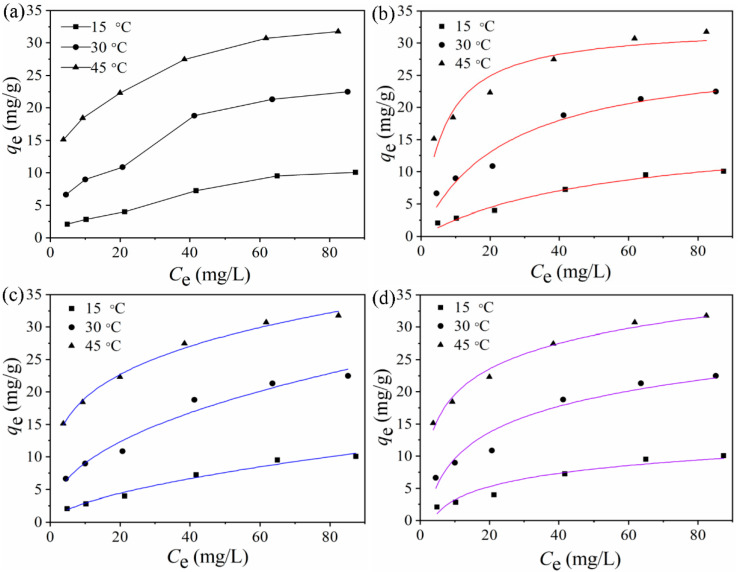
(**a**) Adsorption isotherms for Cd(II) adsorption onto graphene oxide at different initial concentration; (**b**) Langmuir model; (**c**) Freundlich model; (**d**) Temkin model: *m*/*V* = 84 mg/L, *t* = 24 h, pH = 6.00 ± 0.01, *T* = 15, 30, 45 °C.

In actual wastewater, the concentration of cadmium ions can vary greatly. In order to analyze the adsorption capacity of MGO to cadmium ions at different initial concentrations, the effects of temperature on the adsorption capacity of cadmium ions at different initial concentrations were investigated at 15, 30, and 45 °C. The results are shown in Figure 6a. The amount of cadmium ions adsorbed by MGO increased with increasing initial cadmium ion concentration under all three temperature conditions. However, the increase became insignificant and eventually plateaued when the initial cadmium ion concentration reached 60 mg/L. This trend indicates that the adsorption of cadmium ions on MGO reached the equilibrium condition. In summary, the experimental results demonstrate that the amount of cadmium ions adsorbed by MGO varies with the initial cadmium ion concentrations. With a fixed amount of MGO, the total number of active adsorption sites is fixed. Therefore, until saturation, a sufficient number of adsorption sites are always available for the increasing number of cadmium ions in the solution. Consequently, a higher cadmium ion concentration will lead to higher adsorption capacity. Once the adsorption sites are saturated, the adsorption capacity will no longer increase with a further increase in the cadmium concentration.

The adsorption of Cd(II) by magnetic graphene oxide was fitted using the Langmuir model, Freundlich model, and Temkin model at 15, 30, and 45 °C. The fitting results are shown in Figure 6b–d. The relevant parameters used in the models are shown in Table 4. Combining the fitting results with the relevant data, it was found that the Freundlich model yielded the highest correlation coefficient under all the three temperature conditions. Furthermore, the adsorption capacity predicted by this model was the closest to the experimental measurements. These findings show that the Freundlich model can better describe the multilayer adsorption phenomenon of cadmium ions on MGO than the Langmuir and Temkin model, which further suggested that the Cd(II) adsorption could be described as chemisorption on heterogeneous surface. The constant *K*_F_ used in the Freundlich model represents the adsorption capability of the adsorbent. The *K*_F_ values associated with the adsorption of cadmium ions by MGO at 15, 30, and 45 °C is 0.763, 2.309, and 11.78, respectively. This result demonstrates that higher temperatures can promote the adsorption of cadmium ions.

### 3.5. Fractional Factorial Design

**Figure 7:**
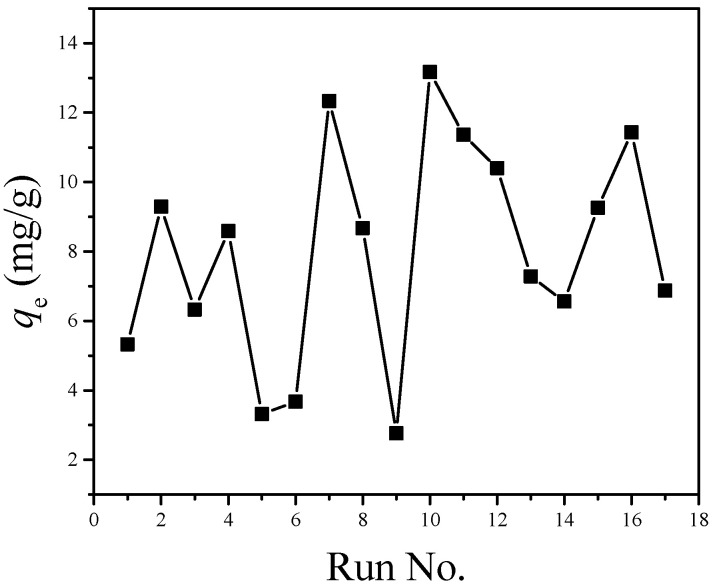
Experimental data obtained from the FFD experiments.

FFD enables people to obtain the same information as a full-factorial experiment through a small number of experiments, thus improving research efficiency [40,41]. The 2^5−1^ FFD with a resolution of V is conducted to analyze the combined influence of five factors (including pH value, temperature, time, initial concentration of cadmium ion, and NaCl concentration) on the adsorption of cadmium ions by MGO. Figure 7 shows the experimental results. When the five factors are all at their intermediate levels (specifically, pH value is 6, temperature is 30 °C, time is 35 min, initial concentration of cadmium ions is 40 mg/L, and NaCl concentration is 55 mM), the adsorption capacity of cadmium ions is 8.674 mg/g (the running order is 8). Cadmium adsorption is affected by different factors in different degrees, and the absorption capacity of cadmium ion is between 2.761 and 13.169 mg/g. This shows that the adsorption process is influenced significantly by the selected factors. The adsorption capacity of cadmium ions under running orders 7 and 10 is larger than that under other running orders. The three factors (including pH value, temperature, and initial concentration of cadmium ions) at the two experimental points are all at high levels. This shows that higher levels of the three factors are beneficial to the adsorption of cadmium ions. At three experimental points (running orders 5, 6 and 9), the pH value and temperature are both at their minimum levels, and the adsorption capacity of cadmium ions were very small.

**Figure 8:**
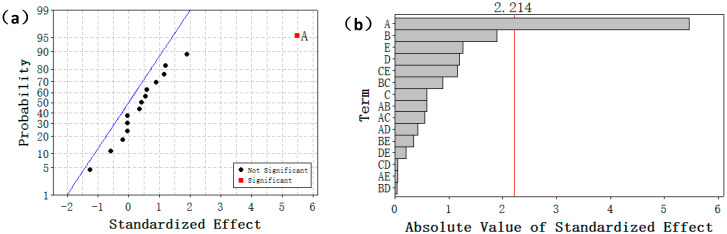
(**a**) Normal plot of the standardized effects; (**b**) Pareto chart of standardized effects for adsorption capacity (qe): (A) pH, (B) temperature, (C) time, (D) Cd(II) concentration, (E) NaCl.

**Figure 9:**
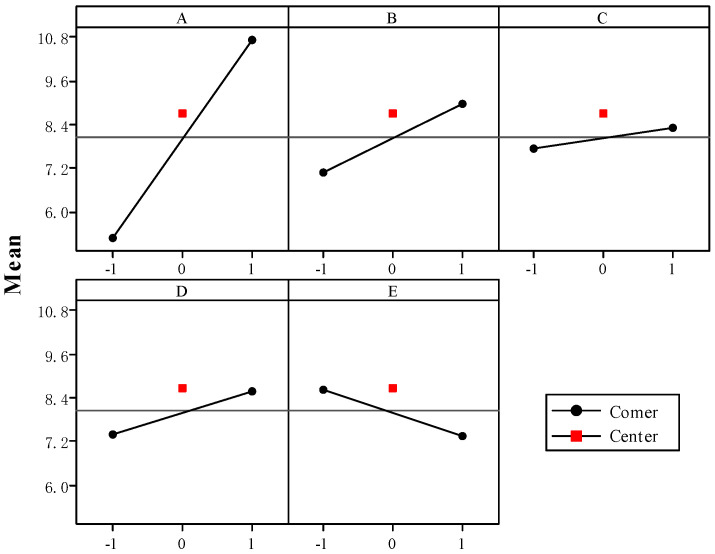
Plots of main effects for Cd(II) adsorption: (A) pH, (B) temperature, (C) time, (D) Cd(II) concentration, (E) NaCl. The symbols −1 and 1 indicate the low and high levels of the factors, respectively.

Figure 8 shows the normal plot and Pareto diagram regarding the influence on the adsorption process exerted by different factors and interactions between them. The Pareto diagram can be used to compare the absolute values of different factors and interactions between them, while the normal plot can be used to analyze the significant influence on the adsorption process exerted by different factors and interactions between them more accurately [42]. As shown in Figure 8a, factor A (pH value) has a significant influence on the adsorption process. As shown in Figure 8b, the adsorption process is influenced by the respective factors in the following order: A (pH value) > B (temperature) > E (NaCl concentration) > D (initial concentration of cadmium ions) > C (time). Figure 9 shows the main effect plot regarding the adsorption of cadmium ions. As shown in this figure, the adsorption capacity of cadmium ions increases most significantly when the level of Factor A goes up gradually. The variations in adsorption capacity arising from the changes in Factors B (temperature) and D (initial concentration of cadmium ions) are more significant than those arising from the changes in Factor C (time). While Factor E (NaCl concentration) increases from 10 to 100 mM, the adsorption capacity of cadmium ions decreases. This shows that the changes in ion strength will reduce the adsorption capacity of cadmium ions, and cadmium ions are transformed into outer-surface complex compounds on the surface of magnetic graphene oxide [43]. Figure 8b also shows that the two interactions (including Factors C and E, and Factors B and C) will influence the adsorption process most significantly.

**Figure 10:**
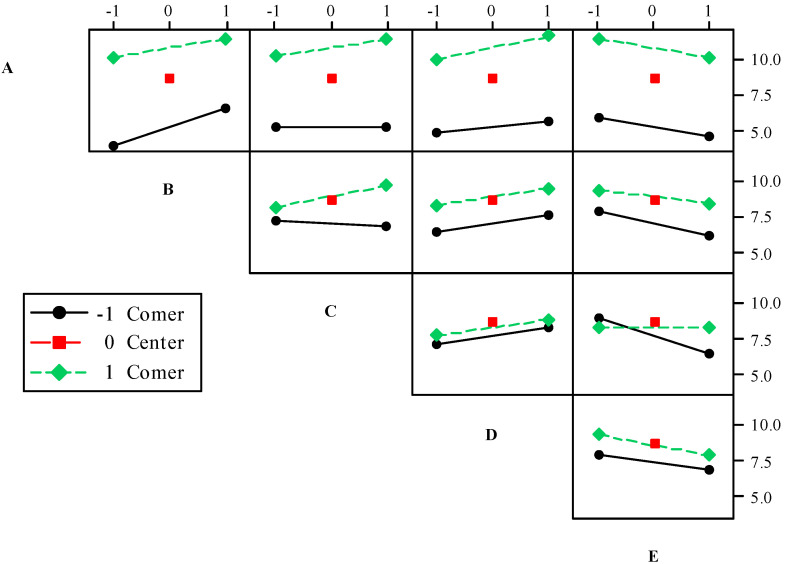
Interaction effects plot for Cd(II) removal: (A) pH, (B) Temperarure, (C) Time, (D) Cd(II) concentration, (E) NaCl.

The interaction diagram is a graph tool used to analyze the average response of all possible combinations of two interactive factors [44]. If the two straight lines in a cell are parallel with each other, it indicates that the interaction of the two corresponding factors is not obvious. If the two straight lines in a cell intersect with each other, it indicates that there is an obvious mutual influence between the two corresponding factors [45]. Figure 10 shows the influence on the adsorption of cadmium ions exerted by the interactions of five factors (pH value, temperature, time, initial concentration of cadmium ions, and NaCl concentration). In the ten cells, the two straight lines in A-E, B-D and C-D are approximately parallel with each other, indicating that the interactions between them are not obvious. In the C-E cell, the included angle composed by the two straight lines is the largest, indicating that Factors C and E have the most significant influence on the adsorption of cadmium ions. The results here are consistent with the results shown in Figure 8b. In addition, Figure 10 shows the changes in the adsorption capacity of cadmium ions when the value of each factor increases gradually. Judging from Row A and Columns B, C and D, the adsorption capacity of cadmium ions goes up with the rise in pH value, temperature, time, and initial concentration of cadmium ions. Judging from Column E, the adsorption capacity of cadmium ions continues to decrease with the rise in NaCl concentration.

## 4. Conclusions

In this work, MGO was synthesized with a variety of active groups on the surface, and Fe_3_O_4_ nanoparticles were successfully loaded on the surface of graphene oxide. The cadmium ions’ adsorption on MGO is remarkably pH-dependent, and the adsorption efficiency improved with the pH value due to the positively charged cadmium (Cd^2+^) ions and negatively charged surface. The sorption kinetics studies illustrated that the kinetics data can be described well with the pseudo-second-order model. The equilibrium data can be well fitted with the Freundlich model. The FFD results show that the adsorption process is influenced by the respective factors in the following order: A (pH value) > B (temperature) > E (NaCl concentration) > D (initial concentration of cadmium ions) > C (time). Among the interactions of the five factors, the two interactions (including time and NaCl concentration, and temperature and time) have the most significant influence upon the adsorption process. In conclusion, MGO is a simple adsorbent with a solid–liquid separation, and represents a potentially cost-effective sorbent for metal ions in wastewater.

## Figures and Tables

**Table 1:** Experimental factors and their values.

Factors		(−)	0	(+)
A	pH	4	6	8
B	*T* (°C)	15	30	45
C	*t* (min)	10	35	60
D	Initial concentration of Cd(II) (mg/L)	10	40	70
E	NaCl (mM)	10	55	100

**Table 2:** Experimental design matrix of the 2^5−1^ fractional factorial design (FFD) with resolution V.

Run	Encoding of Variables				
	A	B	C	D	E
1	−1(4)	1(45)	−1(10)	1(70)	1(100)
2	1(8)	−1(15)	−1(10)	1(70)	1(100)
3	−1(4)	−1(15)	−1(10)	1(70)	−1(10)
4	1(8)	1(45)	−1(10)	−1(10)	1(100)
5	−1(4)	−1(15)	1(60)	−1(10)	−1(10)
6	−1(4)	−1(15)	1(60)	1(70)	1(100)
7	1(8)	1(45)	−1(10)	1(70)	−1(10)
8	0(6)	0(30)	0(35)	0(35)	0(55)
9	−1(4)	−1(15)	−1(10)	−1(10)	1(100)
10	1(8)	1(45)	1(60)	1(70)	1(100)
11	1(8)	−1(15)	1(60)	1(70)	−1(10)
12	1(8)	−1(15)	−1(10)	−1(10)	−1(10)
13	−1(4)	1(45)	1(60)	1(70)	−1(10)
14	−1(4)	1(45)	−1(10)	−1(10)	−1(10)
15	1(8)	−1(15)	1(60)	−1(10)	1(100)
16	1(8)	1(45)	1(60)	−1(10)	−1(10)
17	−1(4)	1(45)	1(60)	−1(10)	1(100)

**Table 3:** Kinetic parameters of Cd(II) sorption on magnetic graphene oxide.

Kinetic Model		5 mg/L	10 mg/L	20 mg/L
		*q*_e,exp_ = 8.50	*q*_e,exp_ = 11.18	*q*_e,exp_ = 15.36
Pseudo-first-order	*k*_1_ (1/min)	0.293	0.339	0.294
	*q*_e,1_ (mg/g)	8.489	10.42	14.66
	*R* ^2^	0.987	0.971	0.952
Pseudo-second-order	*k*_2_ (g/mg min)	0.083	0.077	0.038
	*q*_e,2_ (mg/g)	8.667	10.65	15.13
	*h* (mg/g min)	6.235	8.734	8.699
	*R* ^2^	0.991	0.986	0.982
Elovich model	*α* (mg/g min)	3.01 × 10^12^	1.78 × 10^11^	9.61 × 10^6^
	*β* (g/mg)	4.208	3.095	1.473
	*R* ^2^	0.964	0.981	0.993

**Table 4:** Langmuir, Freundlich and Temkin isotherm parameters for Cd(II) adsorption on magnetic graphene oxide.

Model	Parameter	*T* (°C)		
		15 °C	30 °C	45 °C
		*q*_e,exp_ = 10.35	*q*_e,exp_ = 22.46	*q*_e,exp_ = 31.94
Langmuir	*q*_max_ (mg/g)	16.68	28.43	31.45
	*K*_L_ (L/mg)	0.019	0.45	0.233
	*R* ^2^	0.971	0.931	0.830
Freundlich	*n*	1.700	3.460	4.347
	*K*_F_	0.763	2.309	11.78
	*R* ^2^	0.972	0.957	0.990
Temkin	*a*_T_ (L/g)	0.308	0.608	5.928
	*b*_T_ (kJ/mol)	0.816	0.448	0.519
	*R* ^2^	0.907	0.918	0.966
